# A novel twelve-gene signature to predict neoadjuvant chemotherapy response and prognosis in breast cancer

**DOI:** 10.3389/fimmu.2022.1035667

**Published:** 2022-10-19

**Authors:** Jin Wu, Yuan Tian, Wei Liu, Hong Zheng, Yuanyin Xi, Yuzhao Yan, Ying Hu, Bin Liao, Minghao Wang, Peng Tang

**Affiliations:** ^1^ Department of Breast and Thyroid Surgery, Southwest Hospital, Army Medical University, Chongqing, China; ^2^ Department of General surgery, Linyi People’s Hospital, Linyi, China; ^3^ Department of Thoracic Surgery, Xinqiao Hospital, Army Medical University, Chongqing, China; ^4^ Department of Neurosurgery, Chongqing General Hospital, Chongqing, China

**Keywords:** breast cancer, tumor microenvironment, immunosuppressive, prognosis, neoadjuvant chemotherapy

## Abstract

**Background:**

Accurate evaluation of the response to neoadjuvant chemotherapy (NAC) provides important information about systemic therapies for breast cancer, which implies pharmacological response, prognosis, and guide further therapy. Gene profiles overcome the shortcomings of the relatively limited detection indicators of the classical pathological evaluation criteria and the subjectivity of observation, but are complicated and expensive. Therefore, it is essential to develop a more accurate, repeatable, and economical evaluation approach for neoadjuvant chemotherapy responses.

**Methods:**

We analyzed the transcriptional profiles of chemo-resistant breast cancer cell lines and tumors of chemo-resistant breast cancer patients in the GSE25066 dataset. We preliminarily screened out common significantly differentially expressed genes and constructed a NAC response risk model using LASSO regression and univariate and multivariate analyses. The differences in bioinformatic features of tumor cells, immune characteristics, and prognosis were compared between high and low-risk group. The potential drugs that could reverse chemotherapy resistance in breast cancer were screened by the CMap database.

**Results:**

Thirty-six genes were commonly up/down-regulated in both NAC chemo-resistant tumors and cells compared to the sensitive tumors and wild-type cells. Through LASSO regression, we obtained a risk model composed of 12 genes. The risk model divided patients into high and low-risk groups. Univariate and multivariate Cox regression analyses suggested that the risk score is an independent prognostic factor for evaluating NAC response in breast cancer. Tumors in risk groups exhibited significant differences in molecular biological characteristics, tumor-infiltrating lymphocytes, and immunosuppressive molecule expression. Our results suggested that the risk score was also a good prognostic factor for breast cancer. Finally, we screened potential drugs that could reverse chemotherapy resistance in breast cancer.

**Conclusion:**

A novel 12 gene-signature could be used to predict NAC response and predict prognosis in breast cancer.

## Introduction

Currently, breast cancer has the highest incidence rate among all cancers worldwide (1). Neoadjuvant chemotherapy (NAC) refers to systemic cytotoxic drug treatment before surgery or radiotherapy and is considered the standard treatment regimen for patients with locally advanced or inoperable breast cancer (2, 3). Accurate evaluation of tumor response to NAC provides important information about tumor biology and prognosis and guides further therapies (4–6). In addition to clinical and pathological evaluation criteria, gene expression signatures have been developed to predict response to NAC (7, 8). Different multi-gene expression signatures, such as genomic grade index (GGI), MammaPrint, and Oncotype DX, have been shown to outperform classic histopathological variables and represent an important step towards personalized breast cancer treatment (9–11). In particular, gene profiles overcome the drawbacks of the relatively limited detection indicators of the classical pathological evaluation criteria and the subjectivity of observation (12).

GGI is a gene expression signature developed to ameliorate histologic grade assessment and to predict the response to chemotherapy (7). Using residual cancer burden index, which is a more accurate pathological evaluation method used as a control, researchers studied 229 postoperative tumor samples from patients who had received NAC (paclitaxel, fluorouracil, doxorubicin, and cyclophosphamide). The higher the GGI values, the better the tumor response to chemotherapy (10, 13, 14). It is noteworthy that, unlike pathological evaluation, GGI assessment is more reproducible. However, it involves a large number of genes (97 genes), resulting in high detection costs and difficulty in clinical application.

**Figure 1:**
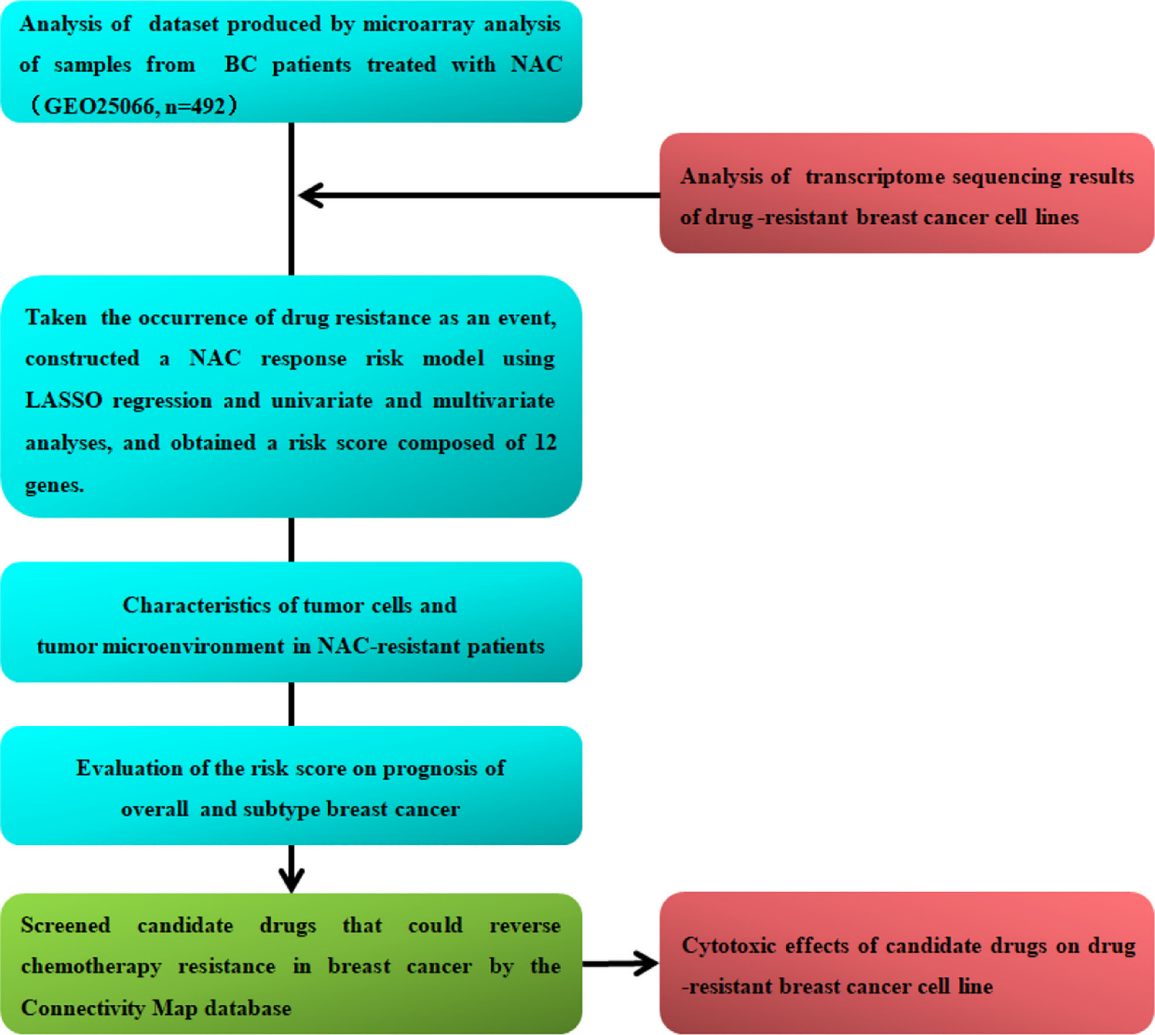
Detailed flow chart of this research.

In this study, we analyzed the transcriptional patterns of breast cancer cell lines and tumors of NAC-resistant patients predict by GGI and screened candidate genes associated with chemoresistance. Furthermore, we constructed a NAC response risk model and examined the evaluation accuracy of the risk score for NAC response. We analyzed the gene expression characteristics, tumor-infiltrating lymphocytes, and immunosuppressive molecule expression of NAC-resistant cancer cells and explored potential drugs to reverse breast cancer chemotherapy resistance. Finally, we examined the risk score for predicting the prognosis of overall and the different molecular subtypes of breast cancer. The flow chart of this research is shown in **Figure 1**.

## Materials and methods

### Cell cultures and chemo-resistant cell line induction

Human breast cancer cell lines MCF-7 (luminal subtype), SKBR3 (HER2+ subtype), and MDA-MB-231 (triple-negative subtype) were purchased from Fu Heng Biology. MCF-7 cells were cultured in DMEM (Gibco, USA) with 10% fetal bovine serum (FBS; Gibco, USA), penicillin (100 U/mL; Gibco, USA), and streptomycin (100 μg/mL; Gibco, USA). SKBR3 cells were cultured in McCoy’s 5A medium (Gibco, USA) with 10% FBS, penicillin, and streptomycin. MDA-MB-231 cells were cultured in Leibovitz’s L-15 medium with 10% FBS, penicillin, and streptomycin. Cells were incubated in 5% CO_2_ at 37.5°C.

Epirubicin (EPI; s1223, Selleck, CHN)-resistant variants of the human breast cancer cell lines were established by pulse selection. Cells were exposed to the respective maximal inhibitory concentration (IC90) values of EPI for 4 h, once a week for 10 weeks, to obtain resistant variants T47D/EPI, SKBR3/EPI, and MDA-MB-231/EPI (15, 16). The EPI-resistant breast cancer cells were washed with PBS, fully lysed with TRIzol reagent (15596026, Invitrogen, USA) and preserved at -80°C until use.

### Cytotoxicity assay and TUNEL assay

MCF-7, SKBR3, and MDA-MB-231 cells were seeded in 96-well plates at 5,000 cells/well. After the cells adhered completely, gradient concentrations of EPI and docetaxel (DOC) were added. After 72 h, cells were stained with sterile methylthiazolyldiphenyl-tetrazolium bromide (MTT; C0009, Beyotime, CHN) in culture media (1:10) for 2 h at 37.5°C. The absorbance of MTT was detected at 570 nm (17, 18).

MCF-7/EPI, SKBR3/EPI and MDA-MB-231/EPI cells were seeded in a 96-well plate at 5,000 cells/well. After the cells adhered completely, EPI (0.04 μM), DOC (0.01 μM), bambuterol (HY-17501A, MCE; 0.04 μM), pravastatin (HY-B0165A, MCE; 0.37 μM), isocarboxazid (HY-13929, MCE; 10 μM), Imexon (HY-15385, MCE; 0.125μM), temozolomide (HY-17364, MCE; 0.12 μM), axitinib (HY-10065, MCE; 1.11 μM), semaxanib (s2845, Selleck), and crizotinib (HY-50878, MCE; 0.37 μM) were added for 72 h, and cells were stained with sterile MTT in culture media (1:10) for 2 h at 37.5°C. The absorbance of MTT was detected at 570 nm. The drug concentration was the same as that used for the connectivity map (CMap) (19).

TUNEL assay was performed using TUNEL apoptosis assay kit (C1086, Beyotime, CHN). Cells were seeded in 48-well plate and treated with drugs (the same with cytotoxicity assay) for 72 h were fixed with 4% paraformaldehyde, and then permeabilized with 0.3% Triton X-100. TUNEL detection solution was added to the cells. After incubation at 37°C for 1h in the dark, cells washed several times with PBS. After sealing with anti-fluorescence quenching liquid, cells were observed under a fluorescence microscope (200×).

### RNA preparation and RNA-seq

Total RNA from MCF-7, SKBR3, MDA-MB-231, MCF-7/EPI, SKBR3/EPI, and MDA-MB-231/EPI was extracted using TRIzol reagent. Genomic DNA contamination of samples was eliminated by RNase-free DNase I. Thereafter, RNA was assessed using a Nano Photometer^®^ spectrophotometer (IMPLEN, CA, USA) and a Qubit^®^ 2.0 Fluorometer (Invitrogen, USA). The RNA samples were subsequently submitted to Sangon Biotech Co., Ltd. (Shanghai, China). Sequencing libraries were generated using the VAHTSTM mRNA-seq V2 Library Prep Kit (Illumina^®^, USA). Paired-end sequencing of the library was performed using NovaSeq sequencers (Illumina, USA). Gene expression values of the transcripts were computed using the String Tie software (version 1.3.3b).

### Differential gene expression and enrichment analysis

Breast cancer transcriptome and clinical data GSE25066 (n=509) were downloaded from the Gene Expression Omnibus (GEO) database (20). The differential gene expression profiles of NAC-resistant and -sensitive patients were analyzed using the R language package (limma 3.20.9). Gene ontology (GO) and the Kyoto encyclopedia of genes and genomes (KEGG) pathway analysis were applied to annotate the biological functions of differentially expressed genes (DEGs) by the R language package (GO plot, KEGG plot function R). The hallmarks of breast cancer chemo-resistant cells were investigated using gene set enrichment analysis (GSEA) (21, 22).

### Diagnostic model construction and validation

After excluding the patient samples with missing data, a total of 492 samples remained in the dataset and were randomly divided into the training (n=246) and validation sets (n=246). Using the “glmnet” R package, we performed the least absolute shrinkage and selection operator (LASSO) regression analysis (23). The Youden index criterion was defined as the boundary-value or decision threshold corresponding to the maximum Youden index, which is the best classification boundary value (24). This was also applied to select the optimal cutoff. We performed Cox regression analysis using the “survival” R package. The “predict” R package was used to obtain the risk score.

### Bioinformatics analysis

To identify the protein-protein interactions between the positive genes of risk score, we employed the search tool for the retrieval of interacting genes/proteins (STRING) (25). The chord diagram was drawn using the Power BI software. The R package “survival” was used for univariate and multivariate analyses of the age, stage, ER, PR, HER2, the signature-based risk factor score, and grade to assess the correlation of NAC resistance with prognosis (26). The expression of twelve genes in tumor, normal tissues and tumor-adjacent tissues were obtained from integrated center for oncology which based on the cancer genome atlas dateset (TCGA) (27). The survival curves of twelve genes of risk score were obtained from the Kaplan–Meier plot (28, 29). Patients with chemotherapy were eligible in this study. The CMap database was used to identify compounds that were negatively correlated with the input differential gene profile after testing on MCF-7 cells (30, 31).

### Immune cell infiltration analysis

We used CIBERSORT and QUANTISEQ analyses to assess immune cell infiltration within the tumor microenvironment using the GSE25066 database in the different risk groups (4). The online analysis tool hiplot (https://hiplot.com.cn/) was used to analyze the correlation between immunosuppressive molecules and risk scores.

### Construction and assessment of the nomogram

The nomogram was established by the R package “rms”. We evaluated the performance of the nomogram by generating a calibration chart.

### Statistical analysis

Using GraphPad Prism 8.0, data of three independent experiments were presented as mean ± SD for statistical analysis. Student’s t-tests or Mann–Whitney U-tests were performed for comparison between two groups. The chi-square test was used to analyze the categorical variables between two groups. The correlation between the two groups was analyzed using Spearman’s test. P < 0.05 was considered significantly different.

## Results

### Screening of hub genes related to NAC resistance in breast cancer

**Figure 2:**
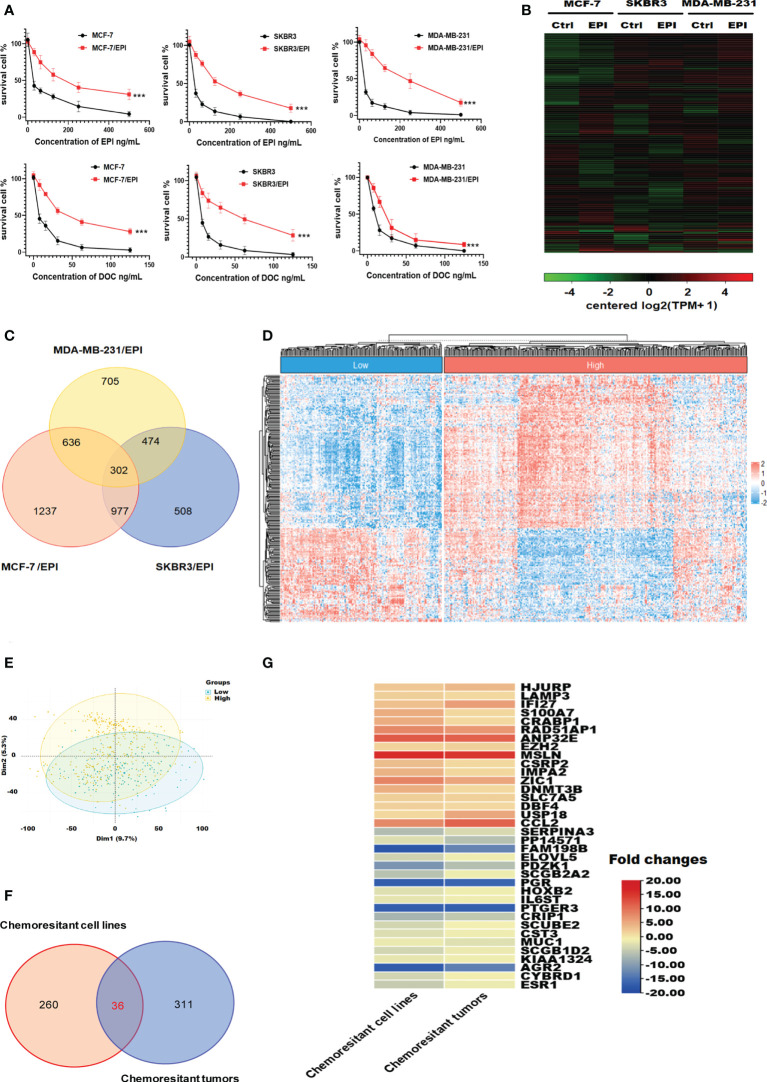
Visualization of genes differentially expressed in chemo-resistant breast cancer cells. **(A)** Drug toxicity of EPI and DOC to resistant cells. **(B)** Heatmaps of DEGs in EPI-resistant cells. **(C)** Overlapping DEGs that were up/down-regulated over 2-fold among EPI-resistant cells lines. **(D)** Heat map of DEGs that were up/down-regulated over 1-fold among chemotherapy-resistant (n = 157) and -sensitive patients with breast cancer (n = 335). **(E)** Principal component comparison of gene expression in chemotherapy-resistant and -sensitive patients with breast cancer. **(F)** Overlapping DEGs among EPI-resistant cells lines and tumors of chemotherapy-resistant patients. **(G)** Heatmaps of commonly up/down-regulated DEGs in both the NAC chemo-resistant cells and tumors compared in contrast to the WT cells and sensitive tumors. Data are presented as mean ± SD (P < 0.001).

To explore the chemoresistance promoting mechanism in breast cancer, we generated EPI-resistant cell lines of different breast cancer subtypes, including MCF-7/EPI, SKBR3/EPI, and MDA-MB-231/EPI. The IC50 of EPI in resistant cells was seven times higher than that of parental wild-type (WT) cells (**Figure 2A** and **Table 1**). Furthermore, consistent with clinical experience, tumor chemoresistance showed characteristics of multidrug resistance in our experiment. Drug-resistant breast cancer cells induced by EPI were also resistant to DOC. We then compared the expression profiles of EPI-resistant cells with those of WT cells. Three hundred and two genes were commonly upregulated or downregulated at least two-fold in MCF-7/EPI, SKBR3/EPI, and MDA-MB-231/EPI compared with those in parental MCF-7, SKBR3, and MDA-MB-231 cells (**Figures 2B, C**). In addition, we analyzed the top 30 GO with the highest enrichment in cellular component (CC), biological process (BP), and molecular function (MF). Upregulated DEGs were widely distributed in the intracellular parts, intracellular organelle, and nucleus of the breast cancer resistant cells, and were enriched in “negative regulation of biological process”, “negative regulation of cellular process”, and “regulation of cellular metabolic process”. The molecular functions of the upregulated DEGs were the “protein and transcription regulatory region DNA” and “RNA polymerase II proximal promoter sequence-specific DNA binding.” In contrast to upregulated DEGs, downregulated DEGs were enriched in the extracellular space of the breast cancer resistant cells in “cell adhesion” and “regulation of cell motility” (**Supplementary Figure S1A–F**). The results showed that slowing down the cell cycle and decreasing biological processes and metabolic abnormalities are important mechanisms for the survival of chemo-resistant cells. In the KEGG enrichment analysis, upregulated chemoresistance cell feature genes were enriched in the “HIF-1 signaling pathway”, “Pentose phosphate pathway”, “p53 signaling pathway, and “DNA replication”. Downregulated chemoresistance cell feature genes were enriched in “Metabolic pathways” and “Neurotrophin signaling pathways” (**Supplementary Figures S1G, H).** The enrichment of these functions suggested that breast cancer chemo-resistant cells may resist chemotherapy by slowing down the cell cycle and strengthening DNA repair and synthesis. To characterize more comprehensively the biological characteristics of breast cancer drug resistance, we analyzed the hallmarks of breast cancer chemo-resistant cells and tumor tissues of breast cancer chemo-resistant patients by GSEA. E2F and MYC targets, mTORC1 signaling, P53 pathway, and KRAS signal gene sets were significantly upregulated in resistant breast cancer cells (**Supplementary Figure S2**), suggesting that chemoresistance of breast cancer cells might be closely related to the regulation of the cell cycle and apoptosis.

**Table 1:** EPI IC50 values in breast cancer cell lines and their chemoresistant variants (n = 3).

IC50 values (ng/mL)	WT	EPI resistance	Fold
MCF-7	25.37 ± 1.93	235.93 ± 6.27	9.30
SKBR3	15.66 ± 0.86	120.27 ± 3.24	7.68
MDA-MB-231	12.53 ± 0.42	171.26 ± 3.73	13.67

Results are expressed as Mean ± S.D. and represent the average of three independent experiments. Fold resistance of each variant is shown in bold and represents the IC50 value of the variants divided by the IC50 value of the WT cells for each particular drug tested.

We also introduced clinical data and transcriptome profiles of breast cancer patients undergoing NAC using the GSE25066 database. According to the GGI evaluation method, GSE25066 samples were divided into two groups: GGI-low (NAC resistance, n=157) and GGI-high (NAC sensitivity, n=335) (**Figure 2D**). The results of the principal component analysis indicated that the gene expression differences between the GGI-high and -low groups were significant (**Figure 2E**). There were 347 DEGs between the two groups. Thirty-six genes were commonly up/down-regulated in both the NAC chemo-resistant cells and tumors compared in contrast to the WT cells and sensitive tumors (**Figures 2F, G**).

### Risk model with NAC response was constructed based on GGI level

**Figure 3:**
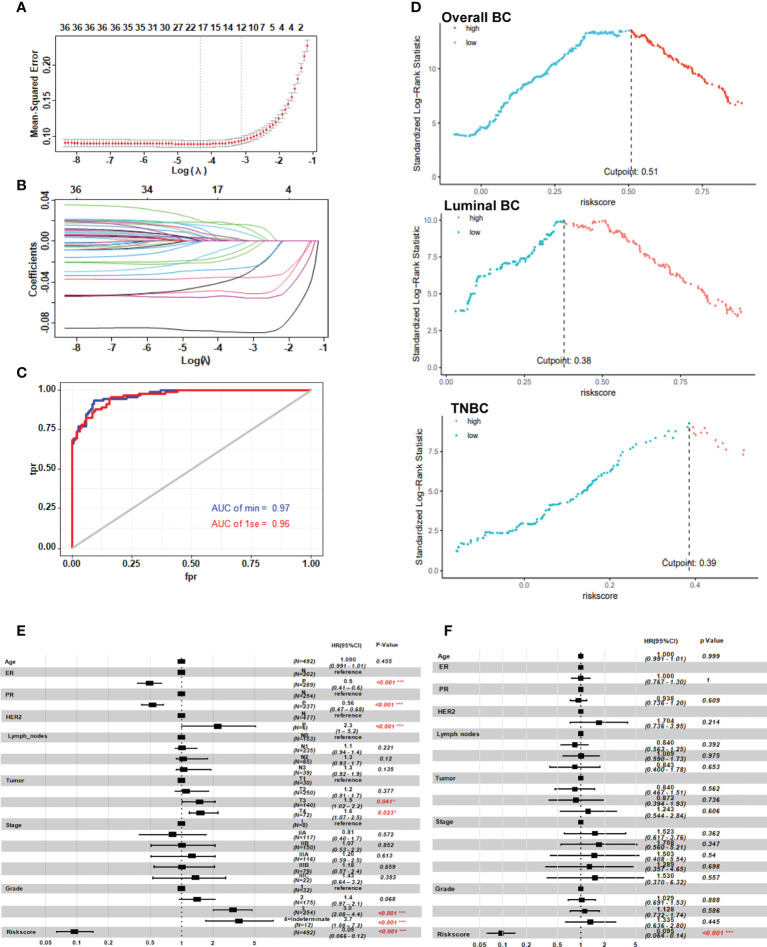
Risk model with neoadjuvant chemotherapy response based on the GGI level between the two clusters. **(A, B)** LASSO regression prognostic model of patients with breast cancer treated with NAC (n=246). **(C)** ROC analysis of the risk score in patients with breast cancer treated with neoadjuvant chemotherapy. **(D)** Cutoff of the risk score in patients with breast cancer treated with NAC. **(E, F)** Univariate and multivariate analysis of the twelve-gene NAC response risk model. *P < 0.05; and ***P < 0.001.

In order to construct a more simplified diagnostic model of NAC response, we established a LASSO regression model based on the expression and prognosis data of 246 breast cancer patients who received NAC from the GSE25066 training set (**Figures 3A, B**). We obtained two gene sets: 1se and min containing 12 and 18 genes, respectively. The ROC analysis presented that the 1se (AUC=0.96) and min (AUC=0.97) of characteristic genes both have good diagnostic values for evaluating the resistance of breast cancer to NAC (**Figure 3C**). Considering the cost of detection for the patients, we selected the 1se set: *HJURP, IFI27, RAD51AP1, EZH2, DNMT3B, SLC7A5, DBF4, USP18, ELOVL5, PTGER3, KIAA1324*, and *CYBRD1*. We termed up-regulated genes (*HJURP, IFI27, RAD51AP1, EZH2, DNMT3B, SLC7A5, DBF4 and USP18*) in NAC chemoresistant cells as positive genes and down-regulated genes (*ELOVL5, PTGER3, KIAA1324*, and *CYBRD1*) as negative genes. The validation set indicated that the results were similar to those of the training set (**Supplementary Figure S3**). The complete names and main function of the 12 genes are listed in **Table 2**. It is well known that there are big different in response to chemotherapy in different molecular subtypes of breast cancer. Therefore, we performed risk cutoff fitting analysis for overall and different molecular subtypes of breast cancer in GSE25066. The results showed that the cutoff of overall BC was 0.51. The cutoff of luminal BC and TNBC were 0.38 and 0.39, respectively (**Figure 3D**). Due to the small sample size, cutoff in HER2+ BC was not obtained. The results of multivariate and univariate analyses on the whole of GSE25066 (n=492) suggested that the risk factor score was an indicator for NAC response (**Figures 3E, F**, p<0.001).

**Table 2:** The function of the twelve genes.

Gene	Full name	Function summary
HJURP	Holliday junction recognition protein	a Protein Coding gene related to Cell Cycle, Mitotic and Chromatin Regulation/Acetylation
IFI27	interferon alpha inducible protein 27 like 2	a Protein Coding gene related to RNA polymerase II activating transcription factor binding and lamin binding.
RAD51AP1	RAD51 associated protein 1	a Protein Coding gene related to RNA binding and single-stranded DNA binding
EZH2	enhancer of zeste 2 polycomb repressive complex 2 subunit	The protein encoded by this gene is a member of the Polycomb-group (PcG) family
DNMT3B	DNA methyltransferase 3 beta	The protein encoded by this gene is a DNA methyltransferase which is thought to function in *de novo* methylation, rather than maintenance methylation.
SLC7A5	solute carrier family 7 member 5	a protein Coding gene related to t peptide antigen binding and antiporter activity
DBF4	DBF4 zinc finger	a Protein Coding gene related to nucleic acid binding and enzyme activator activity
USP18	ubiquitin specific peptidase 18	The protein encoded by this gene belongs to the ubiquitin-specific proteases (UBP) family of enzymes that cleave ubiquitin from ubiquitinated protein substrates.
ELOVL5	ELOVL Fatty Acid Elongase 5	This gene belongs to the ELO family and involved in the elongation of long-chain polyunsaturated fatty acids
PTGER3	Prostaglandin E Receptor 3	The protein encoded by this gene is a member of the G-protein coupled receptor family
KIAA1324	estrogen-induced gene 121	estrogen-induced gene
CYBRD1	Cytochrome B Reductase 1	a member of the cytochrome b(561) family that encodes an iron-regulated protein

### Characteristics of tumor cells and tumor microenvironment in high-risk patients

**Figure 4:**
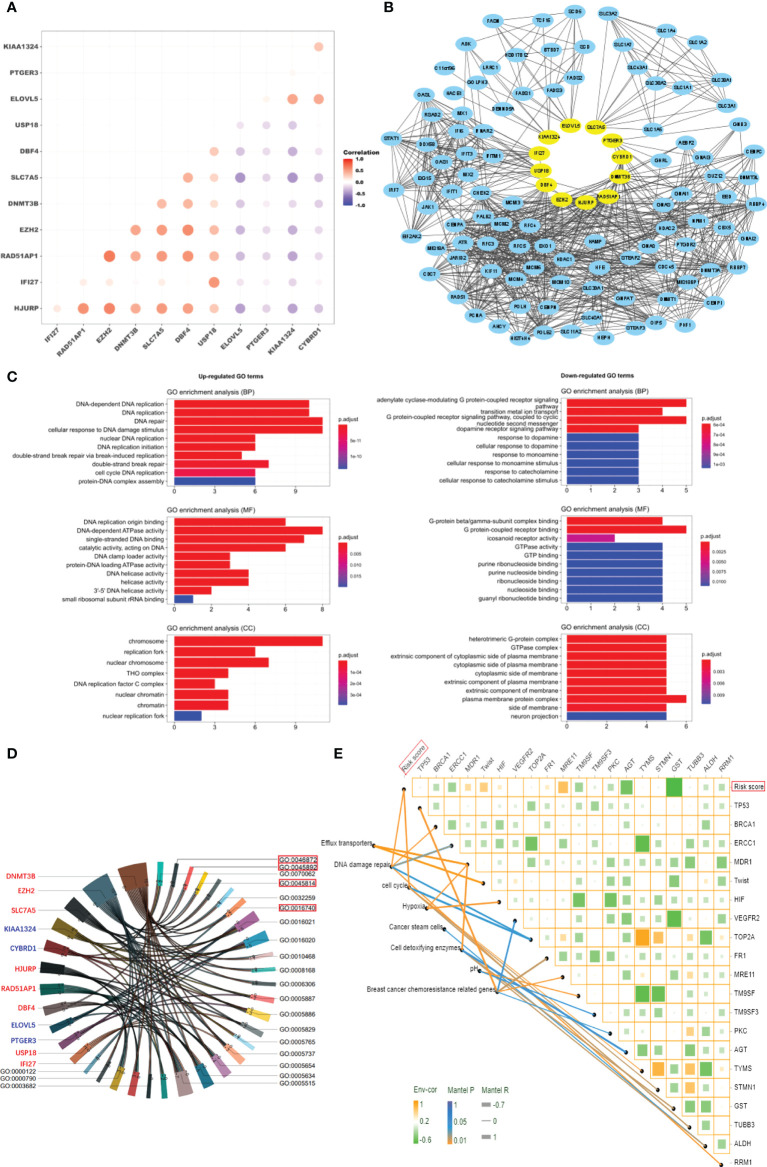
Characteristics of tumor cells in high-risk patients. **(A)** Correlation analysis of the expression of 12 genes. **(B)** Protein network interaction map for the 12 genes. **(C)** GO enrichment analysis of the 12 genes (P < 0.01). **(D)** Chord plot of interaction between the 12 genes and GO enrichment terms. **(E)** The correlation analysis of risk score and gene expression in breast cancer resistance-related pathways.

To explore why 12 genes could be used to predict NAC response, we analyzed pharmacological characteristics and tumor microenvironment of NAC-resistant cancer cells. According to the GSE25066 gene expression profile, a negative correlation was found between the expression of positive genes (HJURP, IFI27, RAD51AP1, EZH2, DNMT3B, SLC7A5, DB*F4 and USP18*) and the expression of negative genes (*ELOVL5, PTGER3, KIAA1324*, and *CYBRD1*) in the risk score model (**Figure 4A**). Utilizing the Hit Predict database, we searched for proteins that interact with those encoded by the above twelve genes. By analyzing their protein interaction network, it was observed that the proteins encoded by these 12 genes and their related proteins have many-to-many complex interactions (**Figure 4B**). The results of GO enrichment analyses suggested that 12 genes promote breast cancer resistance to NAC by upregulating DNA repair and metabolism-related pathways and downregulating membrane receptor signaling mechanisms (**Figure 4** and **Supplementary Table 1**). KEGG pathway enrichment analysis showed that “transition metal ion transport” and the “G protein-coupled receptor signaling pathway” were enriched (**Supplementary Table 1**). The chord plot showed that 12 genes had complex interactions with the enriched GO pathway (**Figure 4D**). For example, *EZH2* and *DNMT3B* were involved in transferase activity (GO0016740) and negative regulation of gene expression (GO0045892 and GO0045814). *DNMT3B, DBF4*, and *CYBRD1* jointly regulate metal ion binding (GO0046872). The above results suggest that these 12 genes promote drug resistance by increasing DNA repair, reducing cell macromolecule synthesis, and cell metabolism among dormant cells. The risk score was positively related to some known molecules (*MDR1, Twist, HIF, MRE11, FR1*) associated with chemotherapy resistance in breast cancer (Xiwei 32). Those molecules were involved in the pathways of cell cycle regulation, DNA repair, transport, and efflux (**Figure 4E**).

**Figure 5:**
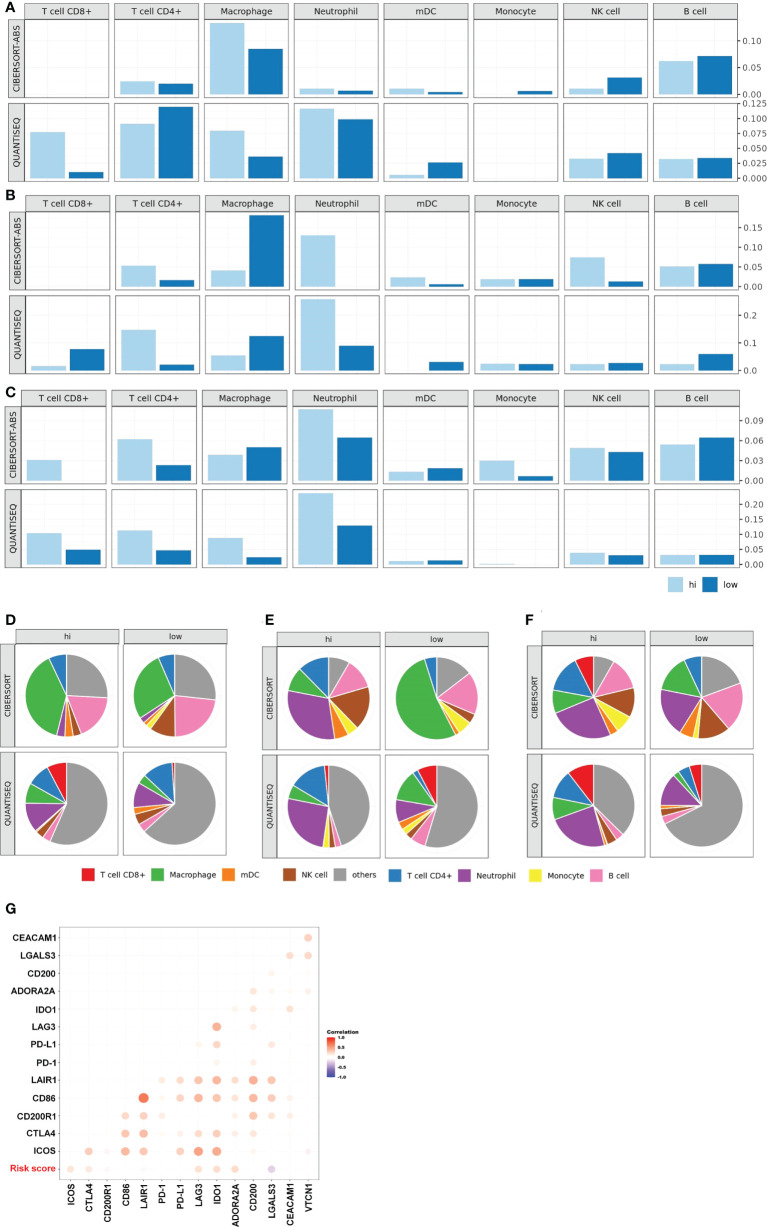
Characteristics of tumor microenvironment in high-risk patients. TIL levels of the high- and low-risk groups in luminal BC **(A)**, HER2+ BC **(B)**, TNBC **(C)**. Proportion of immune cells of the high- and low-risk groups in luminal BC **(D)**, HER2+ BC **(E)**, TNBC **(F)**. **(G)** Heat map of the correlation between risk score and the expression of multiple immunosuppressive regulatory molecules based on the whole GSE25066.

Tumor-infiltrating lymphocytes (TILs) are critical components of the tumor microenvironment and are important external factors of chemotherapy resistance (33–35). Therefore, we assessed the level of immune cell infiltration within the tumor microenvironment of patients in the different risk groups of the GSE25066 database by CIBERSORT, and quanTIseq algorithms. In luminal BC and TNBC, the high-risk group recruited more CD4+ T cell, CD8+ T cell, and macrophage than did the low-risk group (**Figures 5A, C, D, F**). In HER2+ BC, the high-risk group recruited more CD4+ T cell and NK cell than did the low-risk group (**Figures 5B and E**). We further explored the correlation between the risk score and immunosuppressive molecules in the GSE25066 dataset. Many immunosuppressive molecules, such as CTLA4, LAG3, ICOS, IDO1, and ADORA2A, were positively correlated with the risk score (**Figure 5G**). All results indicate that a large amount of tumor-infiltrating cells were depleted in the high-risk group, leading to the failure of NAC for breast cancer and the lower survival rate of patients.

### Evaluation of the risk score on prognosis of breast cancer

Chemoresistance play an important role in tumor relapse, often resulting in metastatic disease and cancer-associated mortality (36).To confirm whether the risk score for evaluating NAC resistance was a good prognostic predictor of clinical outcomes, we conducted the following study. According to TCGA-BRCA data, the expression of the positive genes of risk score (*HJURP, IFI27, RAD51AP1, EZH2, DNMT3B, SLC7A5, DBF4 and USP18*) in tumors was higher than that in normal and tumor-adjacent tissues. The expression of the negative genes (*ELOVL5, PTGER3, KIAA1324*, and *CYBRD1*) in tumors was lower than that in normal and tumor-adjacent tissues (**Supplementary Figure S4**). We also found association of high expression of all positive genes and low expression of all negative genes with poor prognosis of breast cancer (**Supplementary Figure S5**). The above results illustrate that the risk score composed of these 12 genes may also serve as a prognostic factor for breast cancer.

**Figure 6:**
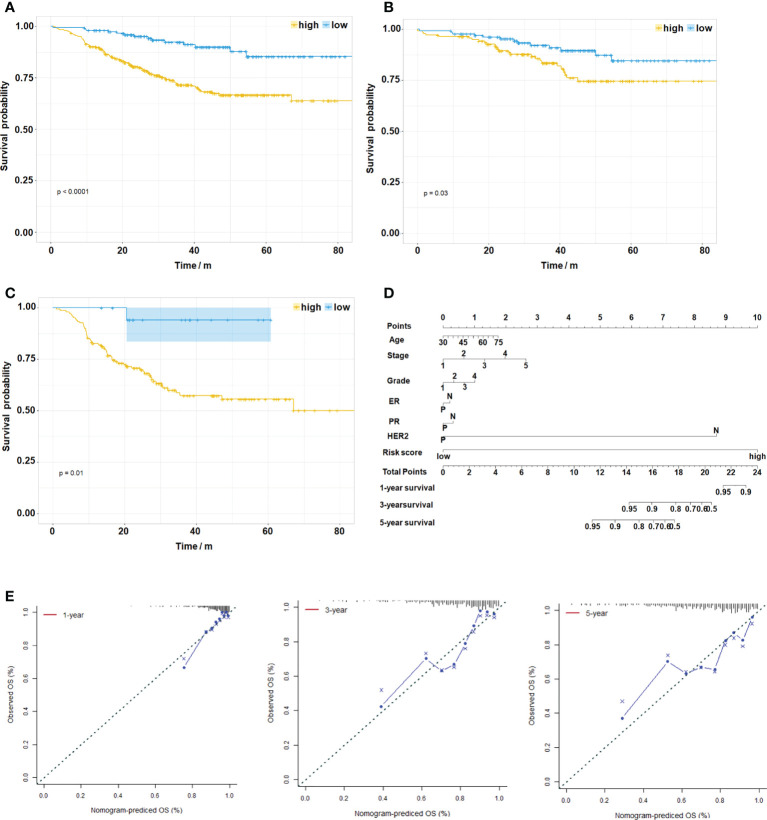
Evaluation of the Risk Score on Prognosis of Breast Cancer. **(A)** Kaplan–Meier survival analysis of the different patient risk groups of overall breast cancer (high-risk, n=333; low-risk, n=159, P < 0.0001). **(B)** Kaplan–Meier survival analysis of the different patient risk groups of luminal BC (high-risk, n=139; low-risk, n=135, P=0.03). **(C)** Kaplan–Meier survival analysis of the different patient risk groups of TNBC (high-risk, n=150; low-risk, n=19, P=0.01). **(D)** A nomogram for clinical diagnosis was constructed based on clinical characteristics and risk scores. **(E)** The calibration plots for predicting recurrence at 1, 3, and 5 years. The X-axis represents the predicted recurrence probability from the nomogram, and the y-axis represents the actual recurrence probability.

As a composite score consisting of 12 genes, can the risk score be used as an evaluation index for prognosis? We analyzed the relationship between risk score and prognosis of breast cancer patients in the GSE25066 dataset. The results suggest that high risk score predicts poor prognosis in overall breast cancer (**Figure 6A**, p<0.0001) and various subtypes (**Figure 6B**, luminal BC, p=0.03; **Figure 6C**, TNBC, p=0.01). Due to the small sample size, statistical test in HER2+ BC was not obtained. Based on the GSE25066 database, we also constructed a nomogram to facilitate clinical application. Using the nomogram, the patient survival probability could be predicted by the weighing age, ER, PR, HER2, grade, stage and signature-based risk score (**Figure 6D**). The calibration curves indicated that the nomogram-predicted probability matched the actual 1-, 3- and 5-years survival (**Figure 6E**). Above results suggest that the novel signature of the 12 genes can not only predict NAC response but also predict prognosis in breast cancer.

### Compound screening for reversing breast cancer resistance

**Figure 7:**
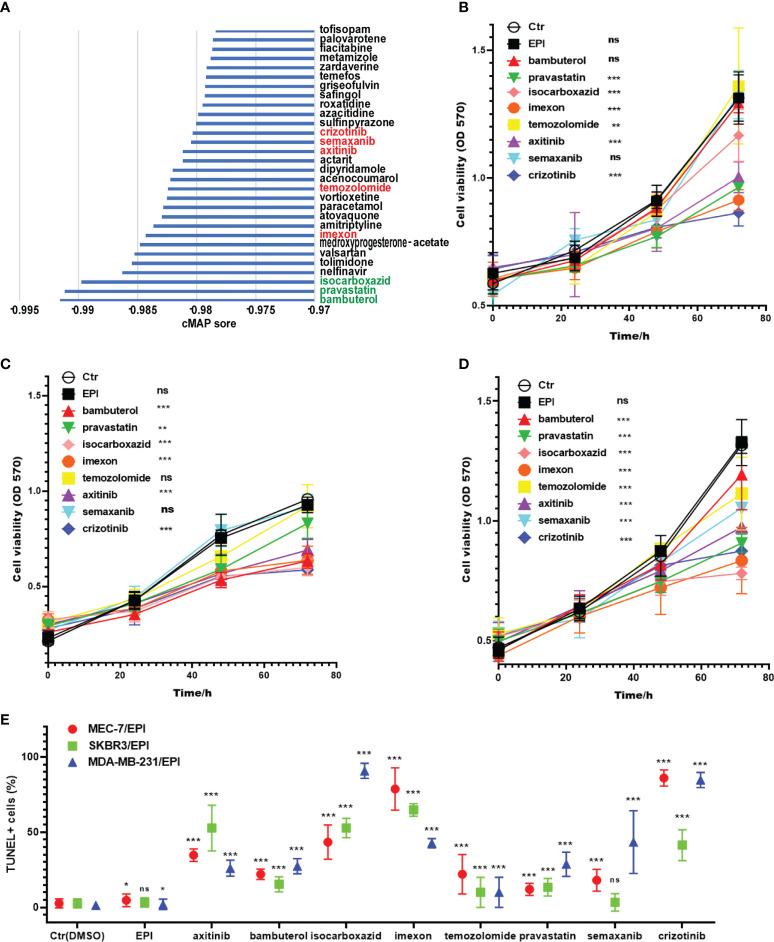
Screening potential drugs for the treatment of high-risk patients. **(A)** The potential drugs for the treatment of high-risk patients. The three best drug candidates are in the green font, and the known anticancer drugs in the TOP30 are in the red font. Cytotoxic effects of preferred drug candidates on MCF-7/EPI **(B)**, SKBR3/EPI **(C)** and MDA-MB-231/EPI **(D)** after 72 h of drug treatment. **(E)** The apoptosis level of cells in each group after 72 h of drug treatment. Data are presented as mean ± SD. *P < 0.05; **P < 0.01; and ***P < 0.001. ns, no significant

To explore the use of candidate drugs to overcome NAC resistance in breast cancer, we analyzed the DEGs of two risk groups. We have screened 169 upregulated DEGs and 92 downregulated DEGs in the high-risk group of GSE25066 (| Fold Change | >1). To screen out the top 30 candidate drugs to overcome NAC resistance in breast cancer, we imported DEGs into the CMap database (**Figure 7A**). The top three compounds were bambuterol (bronchodilator) (37), pravastatin (lipid-lowering agent) (38), and isocarboxazid (antidepressant, a non-selective and irreversible inhibitor of monoamine oxidase) (39). It is noteworthy that there were five anticancer drugs among the candidate compounds, namely, imexon (alkylating agent) (40), temozolomide (alkylating agent) (41), axitinib (inhibitor of tumor growth and phosphorylation of VEGFR-2) (42), semaxanib (VEGFR (Flk-1/KDR) inhibitor) (43) and crizotinib (ATP competitive protein kinase inhibitor met/ALK/ROS) (44). Furthermore, we used the previously established chemoresistant cell line to verify the function of the selected candidate drugs to reverse chemoresistance. Here, we selected the top three compounds and the five antitumor drugs as candidates for verification. Pravastatin, isocarboxazid, imexon, axitinib, and crizotinib had significant cytotoxic effects on MCF7/EPI cells (**Figure 7B**). Bambuterol, isocarboxazid, imexon, axitinib and crizotinib had significant cytotoxic effects on SKBR3/EPI cells (**Figure 7C**). Bambuterol, isocarboxazid, imexon, temozolomide, axitinib, semaxanib and crizotinib had significant cytotoxic effects on SKBR3/EPI cells (**Figure 7D**). The sensitivity of different breast cancer subtypes to these drugs varies widely, but we note that isocarboxazid, imexon, axitinib and crizotinib showed preferable cell killing effects in all three types of breast cancer drug-resistant cells. We also observed the morphology of cells in each group after 72 h of drug treatment. Cells treated with isocarboxazid, imexon, axitinib and crizotinib were swollen, had many protrusions, and tended to die (**Supplementary Figure S6**). Finally, we detected the apoptosis level of cells in each group using a TUNEL apoptosis detection kit. The obtained data were consistent with the previous results showing that groups treated with isocarboxazid, imexon, axitinib, and crizotinib displayed numerous dead cells (**Figure 7E** and **Supplementary Figure S6**). Our results suggest that isocarboxazid, imexon and crizotinib could inhibit or kill chemoresistant cells. This result awaits further validation by more *in vivo* and *in vitro* experiments.

## Discussion

In this study, we constructed a NAC response risk model based on GGI and obtained a novel signature of 12 genes to predict NAC response and predict prognosis in breast cancer. Through pharmacological features analysis, we found that NAC-resistant breast cancer cells have powerful survival strategies, such as cell cycle regulation, DNA repair, transport, and efflux. TME analysis showed that there were many exhausted tumor-infiltrating lymphocytes (TILs) in the tumor tissues of patients in the high-risk group. Another important result of this study is that we screened out potential drugs targeting the gene expression characteristics of patients in the high-risk group through CMap. The cytotoxic effects of these drug candidates were further verified in the self-induced chemo-resistant breast cancer cell line MCF7/EPI. According to the cytotoxicity assays, isocarboxazid, imexon, axitinib, and crizotinib might be potential drugs to inhibit or kill chemo-resistant cells.

Bioinformatics analysis suggested that these 12 genes promote breast cancer resistance to NAC by upregulating DNA repair- and metabolism-related pathways and downregulating membrane receptor signaling mechanisms (**Figures 4C, E**). Based on these characteristics, we found that the NAC-resistant cells in breast cancer might be in a dormant state. Accumulating evidence reveals that non-genetic processes drive drug tolerance, regarded as a novel mechanism of failure in cancer therapy (9, 45). The most recent studies found that as key players in the field of non-genetic heterogeneity of tumors, drug-tolerant persister (DTP) cells were confirmed to be associated with resistance to chemotherapy and targeted agents in a wide range of tumors (46–49). Therefore, DTPs might be a therapeutic opportunity before the patients develop irreversible genetic mutation-driven drug resistance. Here, the 12 genes serve as a potential target for reversing drug resistance of tumor cells by breaking the “cold state” of drug-resistant cells. In follow-up studies, we will investigate whether interfering the expression of these genes could reverse chemotherapy resistance in breast cancer.

The tumor microenvironment plays a crucial role in tumor drug resistance (33–35). Among them, TILs are widely recognized as one of the most promising targets for reversing tumour drug resistance. Our results showed that there were many TILs, such as CD8+ T cells, CD4+, and NK cells, in the high-risk group which were exhausted (**Figure 5**). This also explains why a poor prognosis is observed in the high-risk group although the patients have abundant immune cell infiltration. Such patients may benefit from adoptive cellular immunotherapy. A clinical trial reported a patient with breast cancer who still had extensive metastases after surgery, chemotherapy, and targeted therapy. After 22 months of treatment with TILs, the tumor completely disappeared, and the patient survived (50). Our results also showed that immunosuppressive molecules, such as CTLA4, LAG3, ICOS, IDO1, and ADORA2A, were highly expressed in the high-risk group. Thus, we conjectured that CTLA4 immunosuppressants might contribute to further treatment of these patients.

The results of the cytotoxicity assays showed that the CMap-based selected candidate drugs, pravastatin, isocarboxazid, imexon, axitinib, and crizotinib, have significant cytotoxic effects on MCF7/EPI. Although imexons, axitinib, and crizotinib are known antitumor drugs, their effect on chemoresistant tumors has not yet been reported. Pravastatin and isocarboxazid were originally used to treat hyperlipidemia and depression, but their significant toxic effect on drug-resistant cells suggests that these two drugs may have other mechanisms of action to inhibit tumors. The antitumor activity of these drugs requires further validation.

## Conclusion

Our results suggest that a novel signature of 12 genes can be used to predict NAC response and predict prognosis in breast cancer.

## Data availability statement

The datasets presented in this study can be found in online repositories. The names of the repository/repositories and accession number(s) can be found below: GEO under accession ID: GSE197931.

## Author contributions

PT, MW, and BL conceived the idea and research method of the study. JW, YT, and WL inducted the drug-resistant cell lines and conducted the cytotoxicity assays. JW and BL performed bioinformatics analysis. HZ, YY, and YH collected the samples. JW and YX were responsible for the writing of the manuscript. All authors contributed to the article and approved the submitted version.

## Funding

This work was supported by grants from the National Natural Science Foundation of China (81302315, 82102992), Natural Science Foundation of Chongqing (cstc2021jcyj-msxmX0473), and the Personnel Training Program of Army Medical University (XZ-2019-505-045).

## Acknowledgments

We would like to thank Editage (https://www.editage.cn) for English language editing.

## Conflict of interest

The authors declare that the research was conducted in the absence of any commercial or financial relationships that could be construed as a potential conflict of interest.

## Publisher’s note

All claims expressed in this article are solely those of the authors and do not necessarily represent those of their affiliated organizations, or those of the publisher, the editors and the reviewers. Any product that may be evaluated in this article, or claim that may be made by its manufacturer, is not guaranteed or endorsed by the publisher.

## Abbreviations

BC, breast cancer; BRCA, Breast Invasive Carcinoma; BP, Biological process; CC, Cellular component; CMap, Connectivity map; DEG, Differentially expressed gene; DOC, Docetaxel; DTP, Drug-tolerant persister; EPI, Epirubicin; EP, estrogen receptor; FBS, Fetal bovine serum; GEO, Gene Expression Omnibus; GGI, Genomic grade index; GO, Gene ontology; GSEA, Gene set enrichment analysis; HER2, Human epidermal growth factor receptor 2; KEGG, Kyoto encyclopedia of genes and genomes; LASSO, Least absolute shrinkage and selection operator; MF, Molecular function; PR, progesterone receptor; ROC, Receiver operating characteristic; TIL, Tumor-infiltrating lymphocytes; TCGA, the cancer genome atlas dateset; WT, Wild-type.
