# Supplementary figures and images for: A novel twelve-gene signature to predict neoadjuvant chemotherapy response and prognosis in breast cancer
Source: Front Immunol. 2022 Oct 19;13:1035667. doi: 10.3389/fimmu.2022.1035667 (PMC9629837; doi:10.3389/fimmu.2022.1035667)

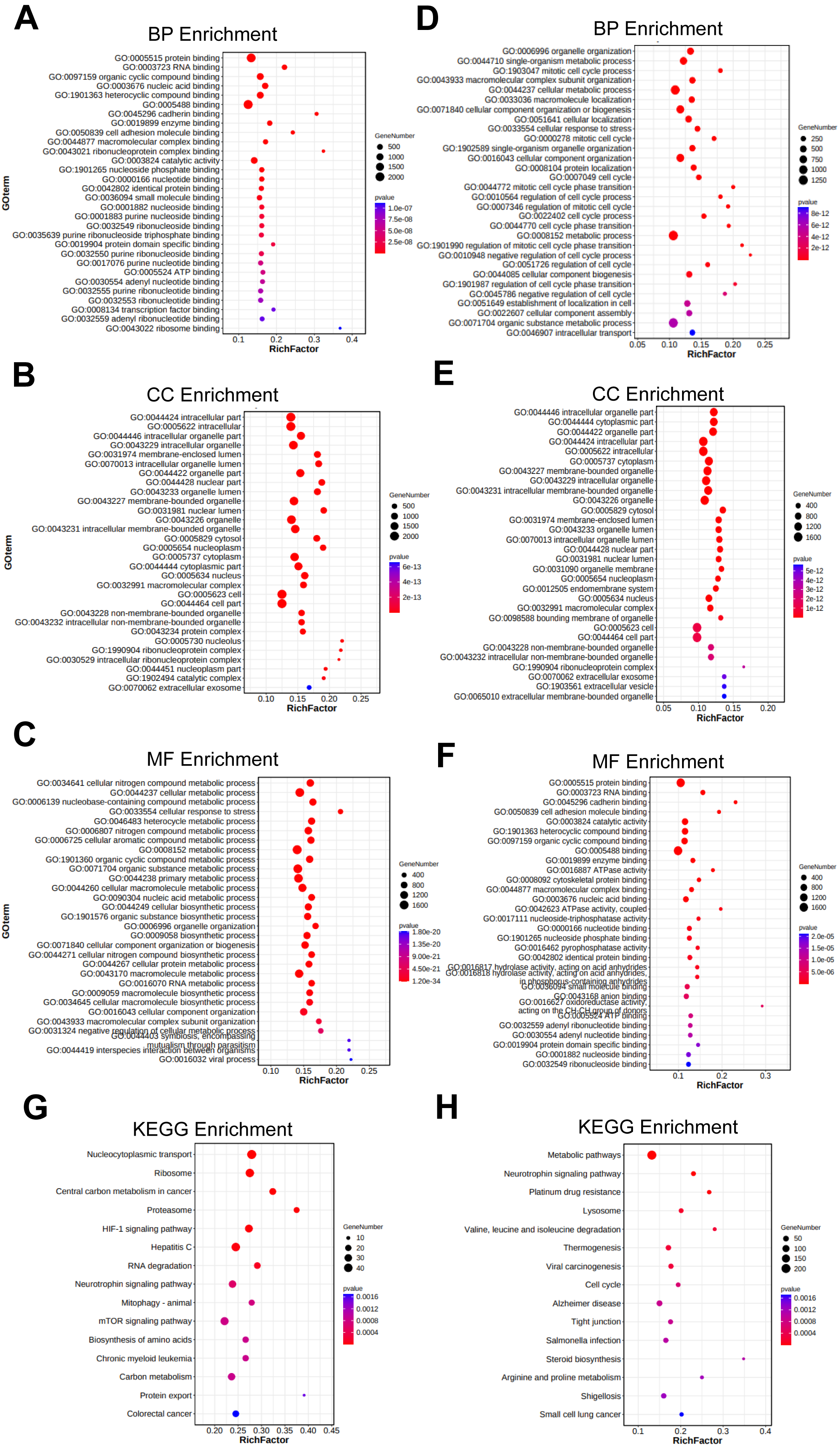

Supplement: Supplementary file 1 [file Image_1.tif]

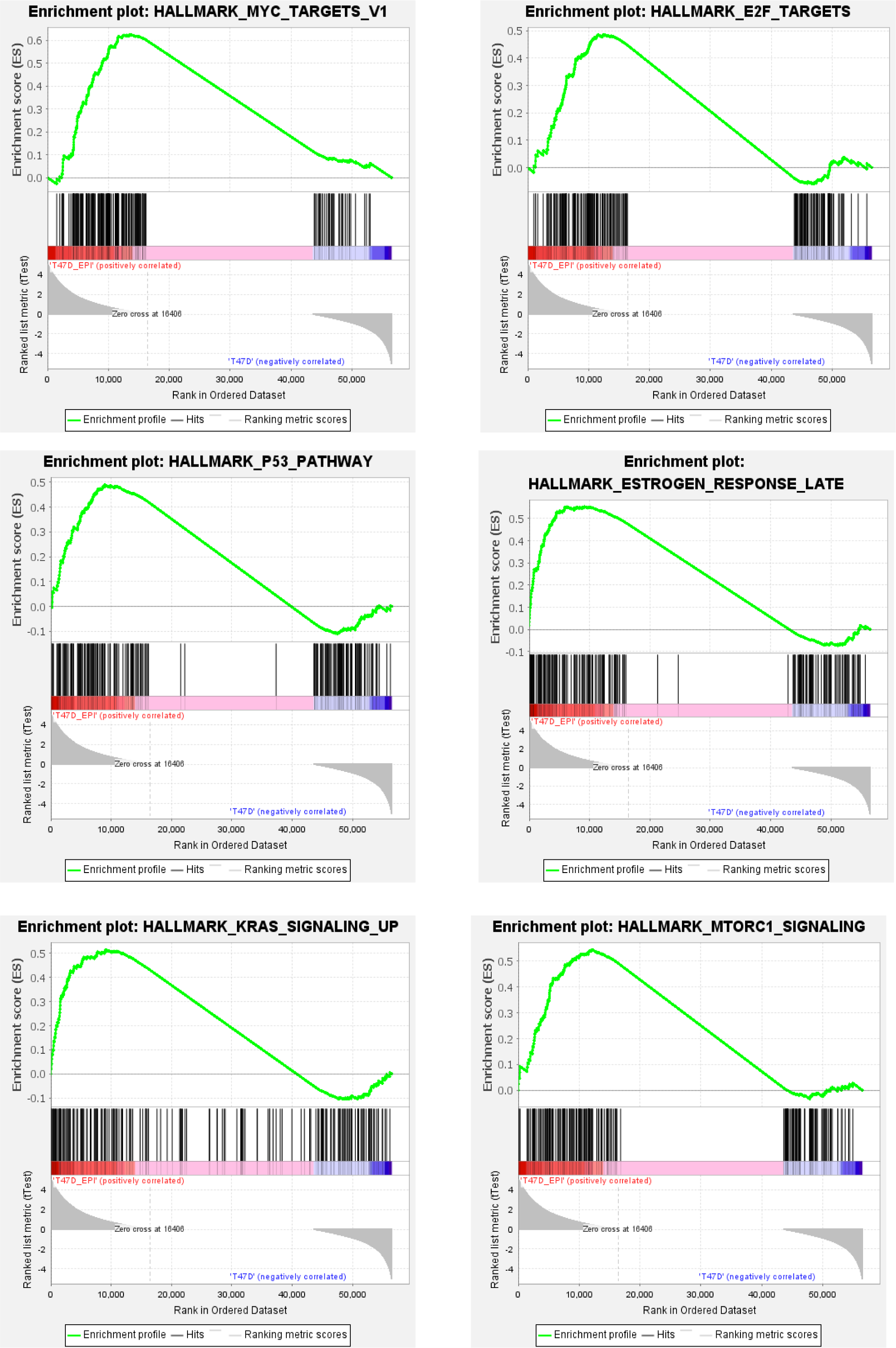

Supplement: Supplementary file 2 [file Image_2.tif]

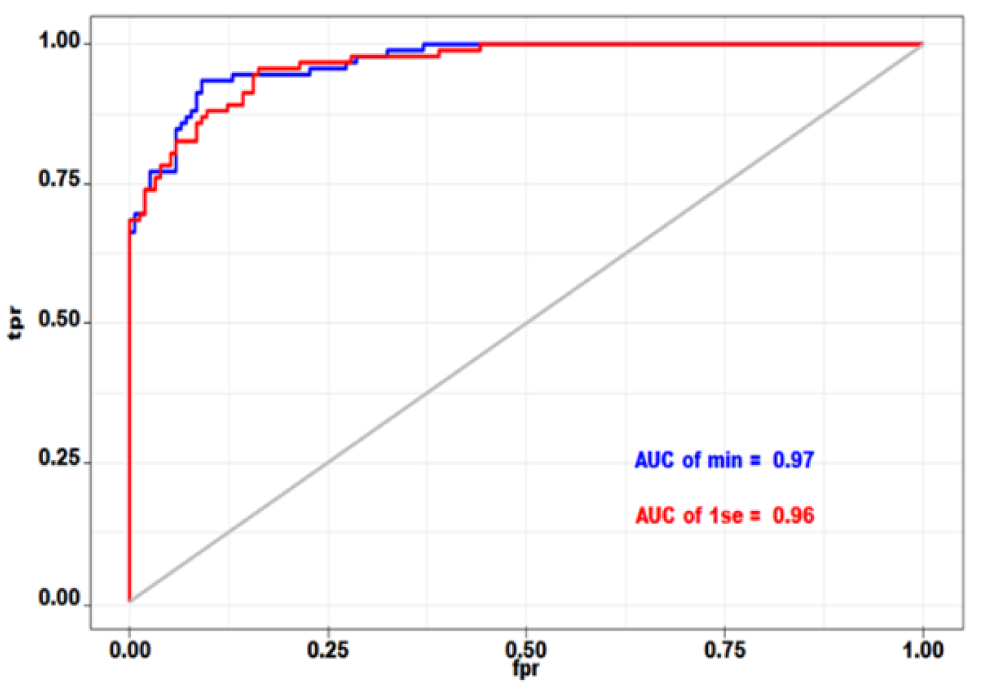

Supplement: Supplementary file 3 [file Image_3.tif]

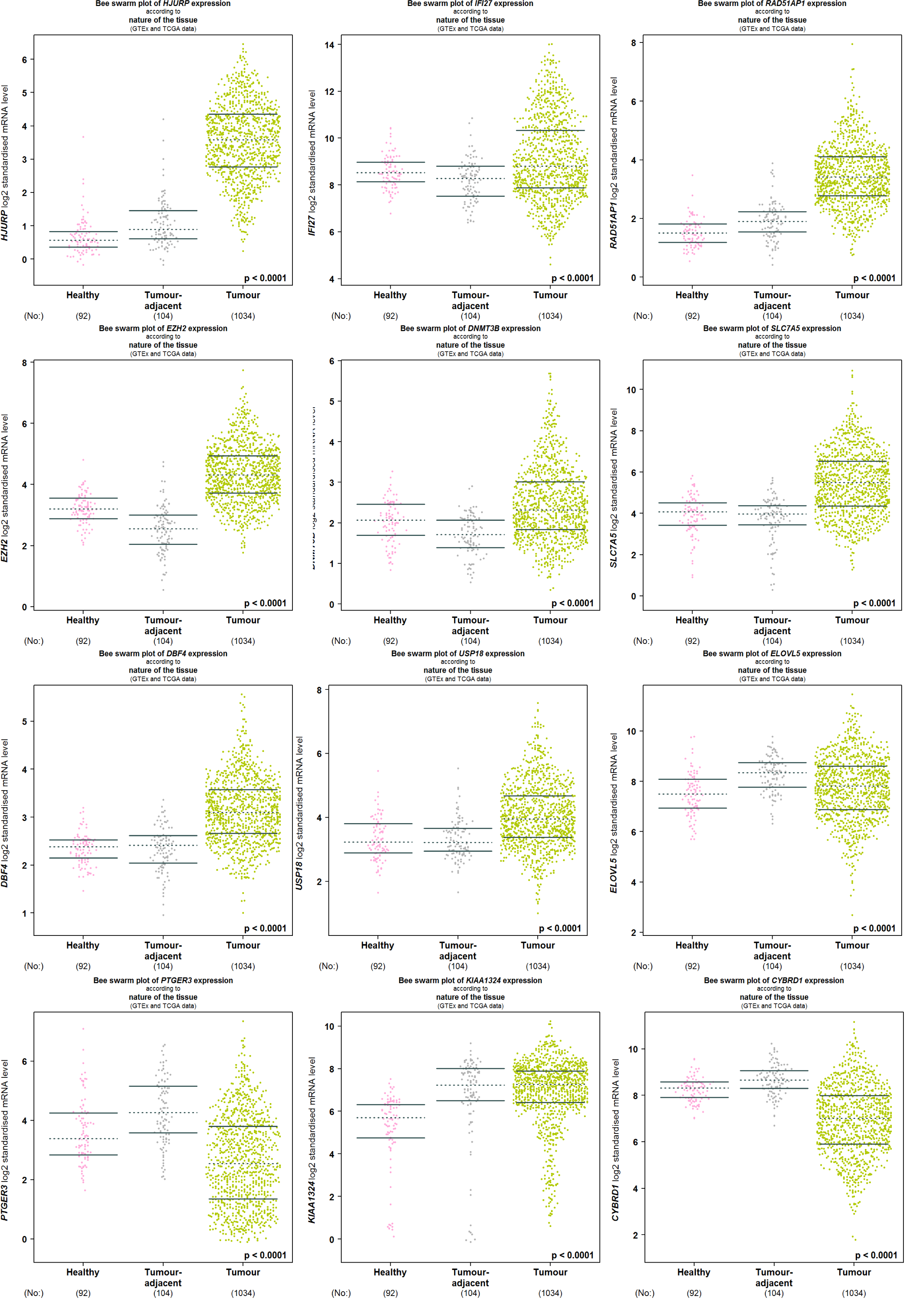

Supplement: Supplementary file 4 [file Image_4.tif]

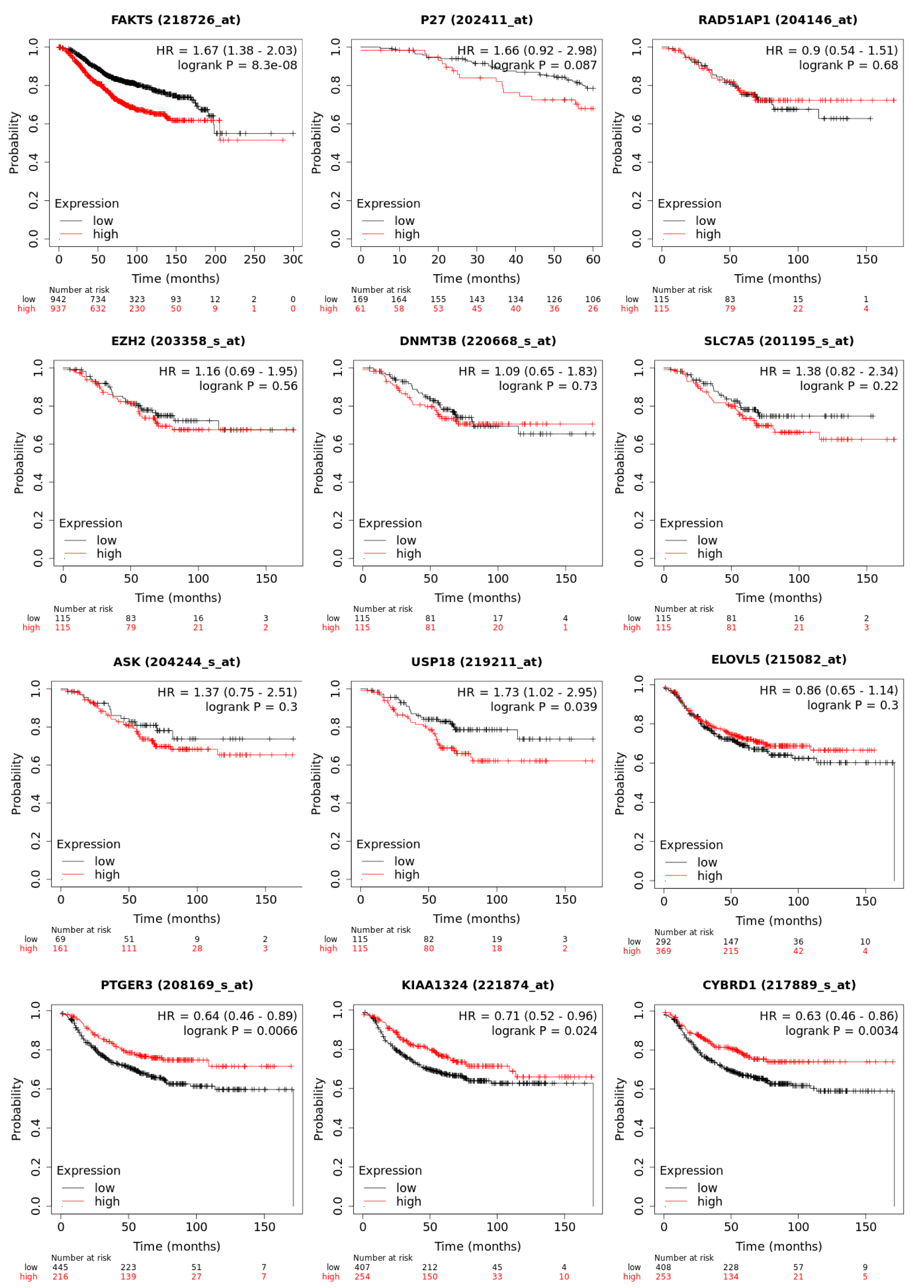

Supplement: Supplementary file 5 [file Image_5.tif]

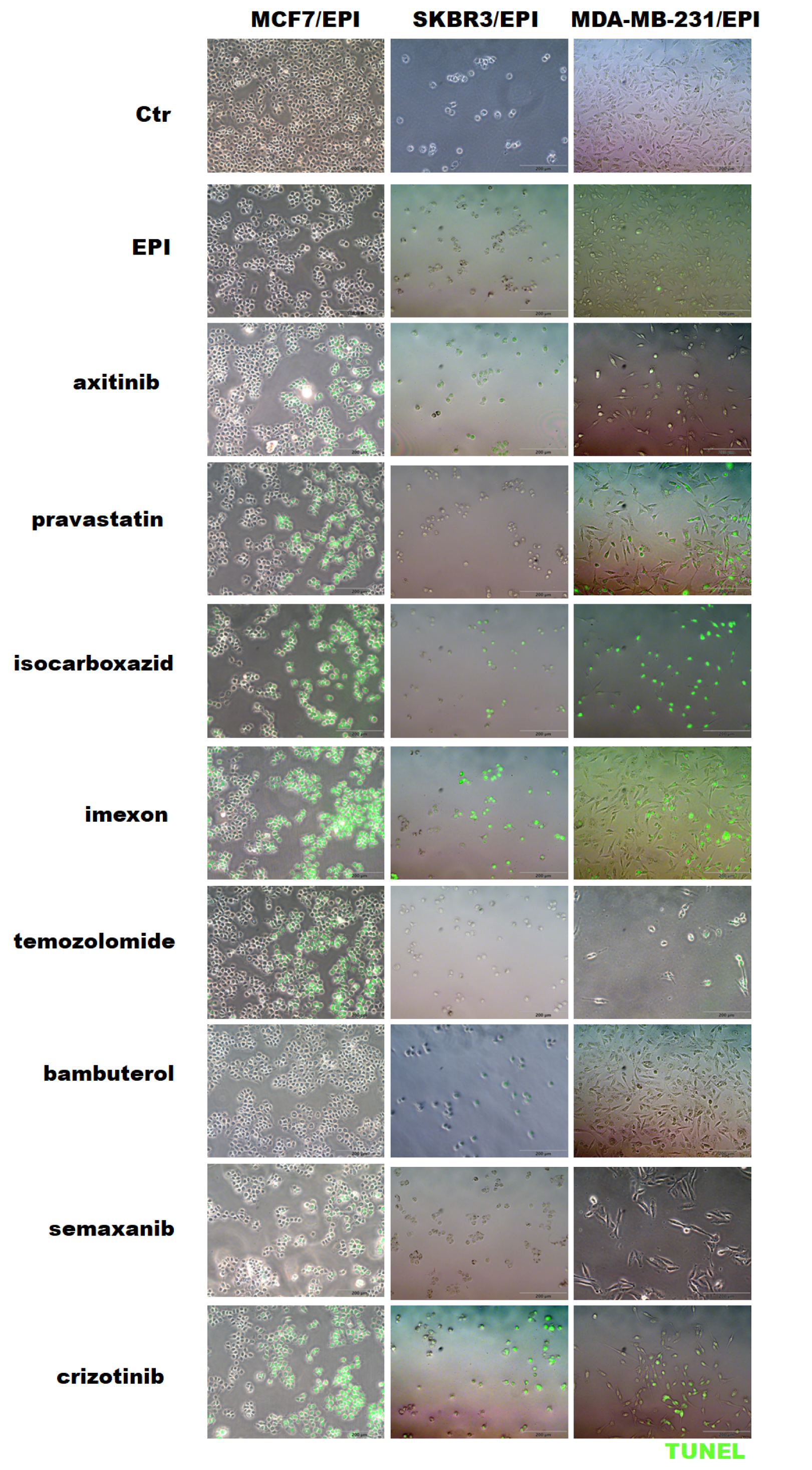

Supplement: Supplementary file 6 [file Image_6.tif]
