# Supplementary material for: A novel twelve-gene signature to predict neoadjuvant chemotherapy response and prognosis in breast cancer
Source: Front Immunol. 2022 Oct 19;13:1035667. doi: 10.3389/fimmu.2022.1035667 (PMC9629837; doi:10.3389/fimmu.2022.1035667)
Supplement: Supplementary file 7 [file DataSheet_1.pdf]

# *Supplementary Material*

**Supplementary Figure S1**

GO analysis of the DEGs in chemotherapy-resistant breast cancer cells. (A~F) up/downregulated GO enrichment terms of EPI-resistant cell lines (P < 0.05). (G, H) up/downregulated KEGG enrichment terms of EPI-resistant cell lines (P < 0.05).

**Supplementary Figure S2**

GSEA of DEGs in EPI-resistant cell lines.

**Supplementary Figure S3**

Obtaining a risk model comprising the same 12 genes in the validation set. ROC analysis of the risk score in patients with breast cancer treated with neoadjuvant chemotherapy. All data are from the randomly selected patients in the GSE25066 dataset as the validation set.

**Supplementary Figure S4**

The expression of twelve genes in tumor, normal tissues and tumor-adjacent tissues.

**Supplementary Figure S5**

Kaplan–Meier survival analysis of twelve genes. (*HJURP* also named *FAKTS*, *IFI27* also named *P27*, and *DBF4* also named *ASK*.)

**Supplementary Figure S6**

The apoptosis level of cells in each group after 72 h of drug treatment (200 ×).

**Supplementary Table 1. the GO and KEGG of twelve-genes**

| Gene name | GO | KEGG |
|---|---|---|
| HJURP | GO:0005515;GO:0005694;GO:0003677;GO:0005739;GO:0005829;GO:0005730;GO:0005634;GO:0007049;GO:0042393;GO:0042802;GO:0007059;GO:0000775;GO:0000777;GO:0005654;GO:0034080;GO:0051101;GO:0043254 | - |
| IFI27 | GO:0016021;GO:0016020;GO:0060135;GO:0034097;GO:0032355 | - |

| | | |
|---|---|---|
| RAD51AP1 | GO:0006281;GO:0003723;GO:0005634;GO:0003690;GO:0003697;GO:0005515;GO:0003677;GO:0006974;GO:0006310;GO:0005654;GO:0010569;GO:0000790;GO:0000724;GO:0000731;GO:0000732;GO:0071479;GO:0036297 | - |
| EZH2 | GO:0018024;GO:0034968;GO:0048511;GO:0005515;GO:0000790;GO:0016569;GO:0031490;GO:0006355;GO:0006351;GO:0032259;GO:0016740;GO:0008168;GO:0005634;GO:0043547;GO:0001047;GO:0000122;GO:0048387;GO:0005654;GO:0003682;GO:0035098;GO:0003677;GO:0045892;GO:0010718;GO:0042752;GO:0045814;GO:0042054;GO:0006325;GO:1990841;GO:0043406;GO:0071902;GO:0016571;GO:0016279;GO:0046976;GO:0070734;GO:0045120;GO:0005737;GO:2000134;GO:1904772;GO:0098532;GO:1900006;GO:0097421;GO:0071168;GO:0070314;GO:0070301;GO:0051154;GO:0050767;GO:0045605;GO:0043433;GO:0042127;GO:0036333;GO:0035984;GO:0034244;GO:0032355;GO:0021766;GO:0021695;GO:0014898;GO:0014834;GO:0014013;GO:0010629;GO:0010468;GO:0006357;GO:0006306;GO:0001932;GO:0070878;GO:0043565;GO:0043021;GO:0003723;GO:0000979;GO:0000975 | K11430-ko05206 MicroRNAs in cancer |
| DNMT3B | GO:0005737;GO:0005634;GO:0071549;GO:0071455;GO:0045892;GO:0045666;GO:0042493;GO:0042220;GO:0033189;GO:0032355;GO:0031000;GO:0014823;GO:0010212;GO:0009636;GO:0001666;GO:0042826;GO:0003682;GO:0003677;GO:0005515;GO:0032259;GO:0016740;GO:0008168;GO:0005654;GO:0046872;GO:0043231;GO:0010468;GO:0006306;GO:0009008;GO:0003714;GO:0000122;GO:0010628;GO:0045814;GO:0051571;GO:0003886;GO:0051573;GO:0090116 | K17399-ko00270 Cysteine and methionine metabolism;ko05206 MicroRNAs in cancer |
| SLC7A5 | GO:0005886;GO:0005829;GO:0016020;GO:0043231;GO:0016021;GO:0015171;GO:0003333;GO:0015807;GO:0015179;GO:1902475;GO:0005737;GO:0006810;GO:0007275;GO:0007399;GO:0030154;GO:0006865;GO:0050900;GO:0005887;GO:0070062;GO:0015297;GO:0016324;GO:0042605;GO:0006520;GO:0015804;GO:0015175 | K13780-ko04150 mTOR signaling pathway;ko05230 Central carbon metabolism in cancer |
| DBF4 | GO:0005515;GO:0043085;GO:0005634;GO:0046872;GO:0008270;GO:0003676;GO:0007049;GO:0006260;GO:0005654;GO:0000082;GO:0008047 | K06629-ko04110 Cell cycle |
| USP18 | GO:0006508;GO:0008234;GO:0008233;GO:0005515;GO:0016787;GO:0016579;GO:0005829;GO:0005737;GO:0005634;GO:0036459;GO:0006511;GO:0004843;GO:0060338;GO:0019785 | - |

| | | |
|---|---|---|
| ELOVL5 | GO:0005783;GO:0042761;GO:0034626;GO:0019367;GO:0006633;GO:0006631;GO:0102338;GO:0102337;GO:0102336;GO:0009922;GO:0006629;GO:0005789;GO:0016740;GO:0016021;GO:0016020;GO:0006636;GO:0034625;GO:0035338;GO:0030425;GO:0042995;GO:0005515;GO:0036109;GO:0043025;GO:0043651;GO:0097447 | K10244-ko01212 Fatty acid metabolism;ko00062 Fatty acid elongation;ko01040 Biosynthesis of unsaturated fatty acids |
| PTGER3 | GO:0007165;GO:0004871;GO:0005886;GO:0007186;GO:0004930;GO:0016021;GO:0016020;GO:0004955;GO:0004957;GO:0005887;GO:0005635;GO:0007200;GO:0031622;GO:0008219;GO:0014827;GO:0060455;GO:0008150 | K04260-ko04020 Calcium signaling pathway;ko04024 cAMP signaling pathway;ko04080 Neuroactive ligand-receptor interaction;ko04923 Regulation of lipolysis in adipocyte;ko05200 Pathways in cancer |
| KIAA1324 | GO:0005886;GO:0005765;GO:0005794;GO:0016021;GO:0016020;GO:0003723;GO:0006914;GO:0005764;GO:0005768;GO:0031902;GO:0070062;GO:0005770;GO:0005887;GO:0005802;GO:0009267;GO:0000045;GO:0044090;GO:2000786 | - |
| CYBRD1 | GO:0005515;GO:0055114;GO:0016491;GO:0046872;GO:0016021;GO:0016020;GO:0005886;GO:0070062;GO:0000293;GO:0010039;GO:0031526;GO:0005765;GO:0006879;GO:0016722 | K08370-ko04978 Mineral absorption |
